# Data Clustering Utilization Technologies Using Medians of Current Values for Improving Arc Sensing in Unstructured Environments

**DOI:** 10.3390/s24134075

**Published:** 2024-06-23

**Authors:** Hee-Jun Kim, Jeong-Ho Kim, Shin-Nyeong Heo, Do-Hyung Jeon, Won-Suk Kim

**Affiliations:** 1 Samsung Heavy Industries Co., Ltd., Geoje-si 53261, Republic of Korea; heejun90.kim@samsung.com (H.-J.K.); snyeong.heo@samsung.com (S.-N.H.); dohyung.jeon@samsung.com (D.-H.J.); 2 Department of Computer Engineering, Pusan National University, Busan 43241, Republic of Korea; touch@pusan.ac.kr; 3 Center for Artificial Intelligence Research, Pusan National University, Busan 43241, Republic of Korea

**Keywords:** arc sensing, DBSCAN, data mining, inverter welder, seam tracking

## Abstract

In the shipbuilding industry, welding automation using welding robots often relies on arc-sensing techniques due to spatial limitations. However, the reliability of the feedback current value, core sensing data, is reduced when welding target workpieces have significant curvature or gaps between curved workpieces due to the control of short-circuit transition, leading to seam tracking failure and subsequent damage to the workpieces. To address these problems, this study proposes a new algorithm, MBSC (median-based spatial clustering), based on the DBSCAN (density-based spatial clustering of applications with noise) clustering algorithm. By performing clustering based on the median value of data in each weaving area and considering the characteristics of the feedback current data, the proposed technique utilizes detected outliers to enhance seam tracking accuracy and responsiveness in unstructured and challenging welding environments. The effectiveness of the proposed technique was verified through actual welding experiments in a yard environment.

## 1. Introduction

Recently, with the increasing introduction of automation and unmanned technologies for smart yards in the shipbuilding industry, the demand for welding robot-based automation in the steel welding sector, which is the core of ship assembly, has been continuously increasing despite various spatial limitations in the shipyard environment [1,2,3,4]. For automated welding using robots, seam tracking techniques that recognize and continuously track the weld seam are essential, and these techniques can be implemented using various sensor technologies [5]. However, in narrow work spaces, such as small- or medium-sized assembly processes, it may be difficult to install additional sensors for seam tracking. To address this issue, arc-sensing technology that performs seam tracking based on the feedback current generated from the welder was proposed [6,7,8].

**Figure 1:**
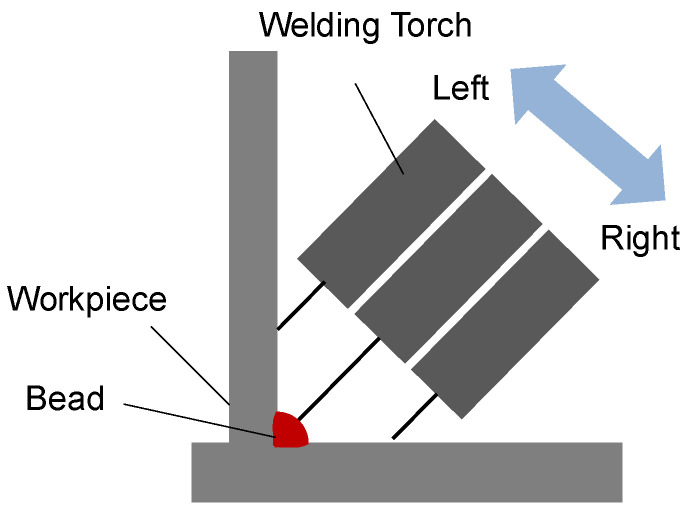
Examples of welding operations with weaving motion.

The current data required for arc sensing are obtained through an ultra-small Hall sensor mounted on the torch cable that sends out the welding wire. The measured feedback current is inversely proportional to the distance between the torch and the welding workpiece. In other words, the current is higher when the torch is closer to the workpiece and lower when it is farther away. Welding is performed through a weaving process, where weaving, as shown in Figure 1, involves moving the torch laterally across the joint in a direction perpendicular to the welding progress, temporarily staying at both ends to fill the bead formed in the weld joint. Thus, due to the changing distance between the workpiece and the torch during weaving, the feedback current also continuously changes. Arc-sensing techniques use this feedback current value to perform seam tracking, continuously correcting the torch’s position [9]. The average current value by area is calculated as the arithmetic mean of each sampled current value.

The weld seams are not always straight and welding environments are not structured. If the distance between the torch and the workpiece is drastically reduced due to severe curvature or a gap exists between workpieces, the feedback current will change rapidly. These drastic changes in the measured current not only reduce the reliability of the sensing data but also cause problems such as large amounts of spatter and very poor welding quality. Therefore, the shipbuilding industry mainly uses inverter welders, which enable precise control with digital signals during arc welding and stabilize the welding current by control of short-circuit transition to reduce spatter when the feedback current suddenly increases [10,11,12]. However, this causes serious problems in the performance of arc-sensing-based seam tracking.

In workpieces with curvature or gaps, inverter welders can stabilize welding performance, but for controlling the secured feedback current values by themselves, the data are not appropriate for use in seam tracking. In other words, the feedback current adjusted due to control of short-circuit transition is drastically lower than the previous current. Accordingly, when applying arc-sensing techniques, the correction of the torch occurs in the opposite direction, leading to a seam tracking failure where the torch gradually moves away from the center of the weld seam.

Moreover, due to the narrow and challenging nature of shipyard work environments, there are cases where straight workpieces are incorrectly recognized as curved workpieces due to the articulation clearance of welding robots, or digital signal loss can occur in the inverter welder due to equipment instability. Under these welding conditions, current control by the inverter welder can occur frequently, leading to a sudden decrease in feedback current and increasing the possibility of seam tracking failure in arc-sensing-based welding automation. Therefore, it is essential to improve arc-sensing techniques to minimize failure in unstructured and challenging environments and to enable accurate seam tracking even in workpieces with curvatures and gaps without additional sensors.

Arc-sensing-based seam tracking algorithms calculate the average current values in the left and right weaving areas using feedback current [13]. By comparing the average values in both weaving areas, they identify the weaving area where the torch is closer to the workpiece and adjust the robot’s offset to move away from that weaving area, thus performing seam tracking. However, in environments with workpieces of large curvature, the distance between the torch and the workpiece can decrease drastically, leading to sudden increase in feedback current followed by a sudden decrease due to control of short-circuit transition. This results in instability in the average feedback current values for each weaving area, ultimately delaying offset correction and causing the torch to go out of the weld seam. Furthermore, even in welding works on workpieces with gaps, if the weaving stay points include the gaps due to delayed correction, sudden changes in feedback current and control of short-circuit transition can occur, increasing the risk of a seam tracking failure.

In this study, in order to solve the above problems related to applying DBSCAN (density-based spatial clustering of applications with noise), a data mining technique, to the feedback current to classify the data and find weaving areas with unstable feedback current [14,15], we propose a new arc-sensing technique that performs seam tracking using data classified as noise. The proposed technique employs the MBSC (median-based spatial clustering) algorithm to classify current data and adjusts the torch correction direction to be closer to the weld seam center when noise data are detected, thus performing seam tracking. This approach improves welding automation robustness by quickly controlling torch bias, reducing seam tracking failure not only in straight welding but also in curved workpieces or gaps between workpieces.

## 2. Related Work

In the field of welding automation, research on seam tracking technology involves various approaches. One approach involves performing measurements before welding and using pre-collected weld seam information for arc welding [16,17]. Xu et al. proposed an approach that analyzes images of the weld seam region created by projecting a circular laser onto the workpiece to pre-conduct seam tracking, and uses this information to automate the welding process. Furthermore, Sung et al. performed seam tracking by projecting multiple straight lasers onto the weld seam region and analyzing the patterns. However, both techniques have the weakness of increasing the overall work time since seam tracking and welding are conducted separately. To address this issue, seam tracking must be performed simultaneously with the welding operation [5].

In this regard, laser shape detection and positioning technology using vision sensors have been primarily utilized [18,19,20]. This method attaches separate laser equipment to shoot a laser beam, which is then measured by a vision sensor. The 2D images captured by the camera are analyzed and converted into 3D for use in seam tracking. Additionally, there are studies that perform seam tracking based on 3D point cloud technology with the converted data [21,22]. Moreover, another technology for seam tracking has been proposed that detects the molten pool (melted metal during welding) using vision sensors [23,24]. However, these methods using a vision sensor face challenges in real-time seam tracking during welding due to noise caused by arc light and spatter, which reduces measurement accuracy. In addition, attaching vision sensors can limit the robot’s range of motion, making it difficult to use in narrow spaces such as shipyard blocks.

Various studies have also been conducted on seam tracking using arc sensing. In particular, several studies have shown that seam tracking techniques using arc sensing, which rely on feedback current without additional sensors, are suitable for the welding environment of shipyard blocks. Research has also been conducted on arc-sensing techniques to overcome cases where gaps exist [25,26,27]. These techniques perform seam tracking using a magnetic-controlled arc sensor and operate robustly under various working conditions, such as straight fillets with variable gaps and welding environments without gaps. However, this technique requires a complex magnetic-controlled arc sensor, which is prone to frequent failures in challenging environments such as shipyard blocks, and the additional sensor can cause usage restrictions in confined spaces.

Separately, studies have been conducted on arc sensing using CNNs (convolutional neural networks), which maintain high accuracy even under fluctuating lighting conditions caused by complex image patterns and spatter compared to traditional arc-sensing techniques [28]. However, these studies also failed to considered various conditions such as workpieces with curvature or gaps. In addition, an RGB camera is required to collect image data for CNN application, and a high-performance GPU is necessary for real-time implementation.

Studies on arc sensing for various workpieces, such as straight fillets and circular workpieces, have also been conducted [29,30]. However, since these studies were conducted based on a specific form, their application is limited in unstructured conditions where predefined forms are not set. An arc-sensing study has also been conducted for welding in a narrow gap, and shares similarities with this study in that it sets arc-sensing conditions for workpieces under challenging conditions [31]. However, this study is limited to very specific conditions, making it difficult to apply arc sensing to workpieces with gaps between them or workpieces with curvature, which are common in shipyards. Considering these points, this study proposes an arc-sensing technique applicable to instances where an abnormal current occurs.

## 3. Proposed Method

### 3.1. Problems of Average Current-Based Arc Sensing

**Figure 2:**
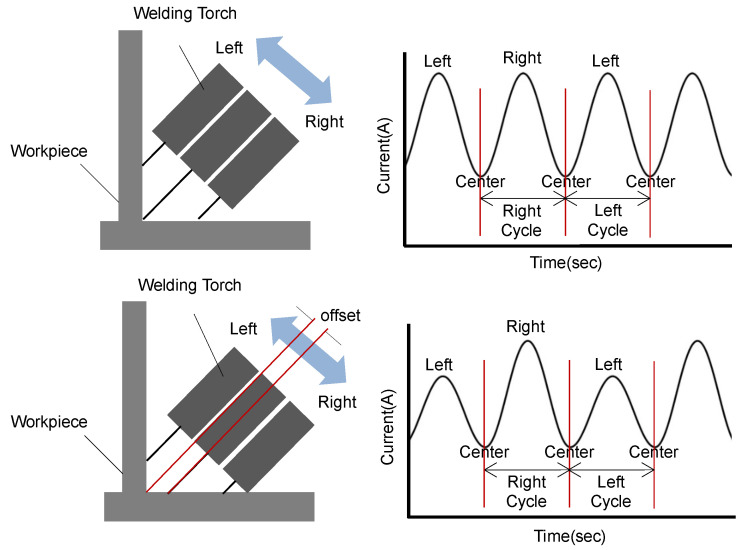
Example of current values in different weaving areas during welding operations.

Arc sensing defines the left and right weaving areas based on the welding center line to take into account the torch’s weaving motion. It uses the average values of the feedback current measured in each area to estimate the torch’s bias. By comparing the average values of feedback current measured in the left and right areas, the area with the relatively higher average value is considered the area where the torch is biased. Figure 2 shows the changes in feedback current due to torch bias. Based on this, an offset is applied to shift the center of the torch. This weaving-area-based average current value arc-sensing technique shows effective performance in large environments, such as when the weld seam is straight. However, when the workpiece has significant curvature, causing rapid changes in the distance between the weld target workpiece and the torch, or when a certain curvature and gap exist on the weld workpiece, the reliability of the feedback current can decrease or the response can be delayed, increasing the possibility of seam tracking failure.

**Figure 3:**
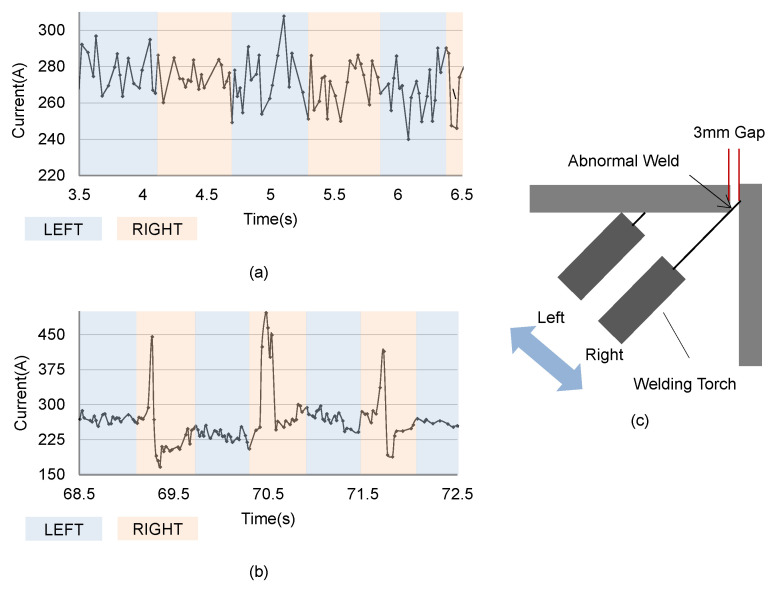
Measurement results of the feedback current values for a workpiece with a certain level of curvature and gap (**a**) when the gap is located at the midpoint of the weaving, (**b**) when the gap is located at the stay point of the weaving, and (**c**) when the gap is located at the stay point of the weaving.

We would like to analyze problem instances by observing the actual feedback current values during the welding of workpieces with a certain level of curvature and gap. Figure 3 shows the weaving-area-specific feedback current values in conditions where there is a 3 mm gap in the target workpiece: (a) when the gap is located at the midpoint of the torch’s weaving, and (b) when seam tracking is delayed and the gap is located at the stay point of the weaving. In condition (a), it can be seen that the feedback current values are collected stably at a certain level. However, in (b), there is a sudden increase in feedback current values followed by a sudden decrease due to the transition to short-circuit control.

During the weaving process, the torch stays at the ends of the left and right weaving areas for a certain cycle before moving to the opposite side. Under normal circumstances, the gap is positioned at the midpoint of the torch’s weaving and passes quickly by without significantly affecting the feedback current values. However, if the offset correction is delayed due to inaccurate and unstable average feedback current values in each area caused by curvature, an instance can occur where the torch stays at the gap point, as shown in Figure 3c. This results in decreased resistance due to the reduced molten pool, causing the feedback current to increase drastically. As mentioned earlier, these sudden high currents can be a major cause of welding defects. Therefore, control of short-circuit transition is performed by the inverter welder, and the measured feedback current shows an abnormal pattern of rapid increases and decreases. In order to alleviate the complexity of the proposed scheme, we assumed general conditions for weld geometry parameters such as weld angle, bead size, and surface characteristics. Based on this, only two scenarios, such as a workpiece with severe curvature and a workpiece with a gap, are adopted for evaluating the effect of the proposed scheme.

**Figure 4:**
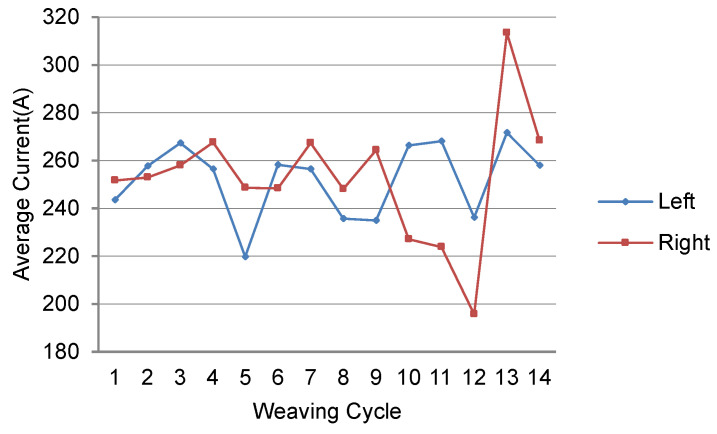
Average feedback current measured under biased welding condition.

Figure 4 shows the weaving-area-specific average feedback current values measured in the same condition as above, displayed for each weaving cycle. Due to the curvature of the workpiece, the torch gradually gets closer to the left side of the workpiece. Therefore, as the weaving progresses, the average current value in the left area should increase, while the average value in the right area should decrease. However, the actual measured average value in the left area does not show much difference even as the weaving progresses and, instead, a sudden large decrease is observed in the fifth cycle, indicating unstable results. Afterwards, in the 11th, 12th, and 13th cycles, corresponding to the part in Figure 3b, very unstable average values are observed in the right area. Moreover, although the torch is closer to the left area and an increased feedback current value should be observed, the average value does not change significantly due to the control of short-circuit transition, which has already lowered the current levels. As a result, the average current value in the left area maintains a certain level, while the average current value in the right area shows very unstable values. According to these actual observations, it can be seen that, in conditions where there is a gap in a curved workpiece, if the offset is not corrected promptly even under the normal state, it can lead to an irrecoverable failure instance like this.

**Figure 5:**
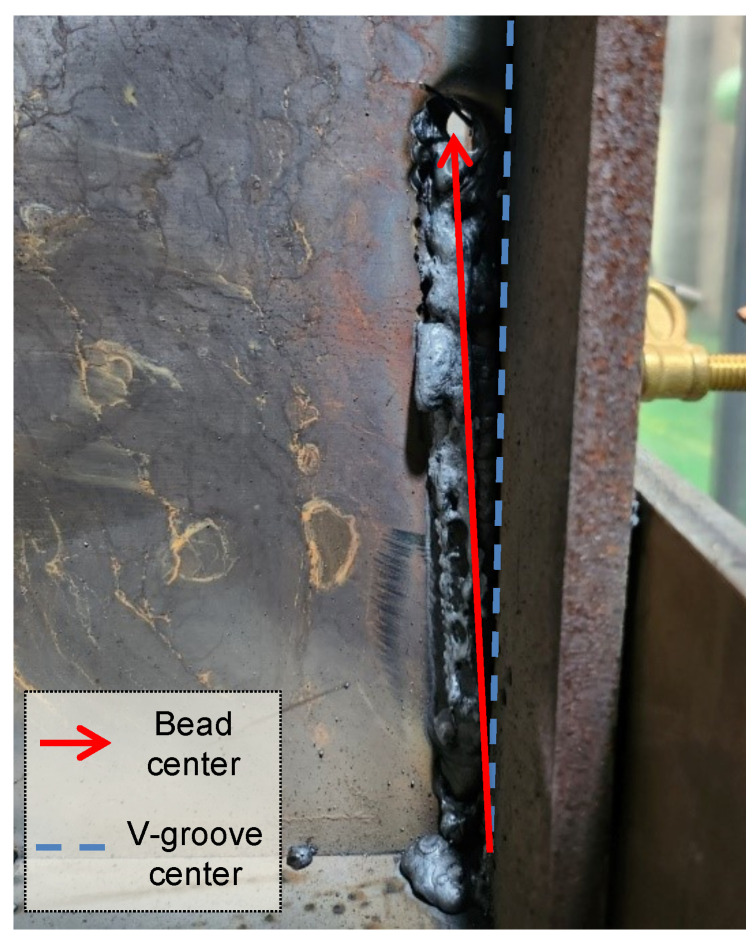
Welding failure in a biased instance.

Figure 5 shows an instance where seam tracking has failed due to the previous reason. According to the formed bead shape, it can be seen that the torch fails to accurately recognize the V-groove center, which is the welding center line, and continues to be biased in one direction. This problem worsens as the welding progresses. As a result, the torch became biased and the welding ends as abnormal due to contact between the torch and the workpiece. The weaving-area-specific average feedback current values can show unpredictable values due to various environmental factors, such as the sampling cycle, sampling success rate, degree of curvature, gap size, and sensor accuracy. In other words, as in the example above, the existence of a gap in a curved workpiece or in workpieces with severe curvature means that the reliability of the area-specific average current values is reduced. Therefore, this study proposes a technique to correct the torch’s position more quickly and reliably through the detection of outliers in the feedback current values.

### 3.2. MBSC-Algorithm-Based Seam Tracking Technique for Automation of Welding in Unstructured Environments

This paper proposes the MBSC algorithm, a technique that enables effective seam tracking based on arc sensing, even in conditions where the feedback current changes drastically due to the control of short-circuit transition. Since actual welding works are carried out under challenging environmental conditions, such as in shipyards, the collection cycle of feedback current data for arc sensing can be quite irregular; that is, despite the feedback current data trend time-series characteristics, the time intervals can be somewhat irregular, making it difficult to apply typical time-series processing techniques such as moving averages. Therefore, the application of a clustering-based algorithm is required to effectively process and analyze irregularly collected data during the welding process.

The proposed technique is based on the DBSCAN clustering algorithm. DBSCAN performs density-based clustering within multidimensional datasets and defines center points based on a given eps (epsilon) value and the minimum minPts (minimum number of points to form a cluster), and then connects them to form clusters. This method, when eps and minPts are appropriately set, can effectively detect outliers in datasets with varying shapes and sizes of different clusters. The proposed technique uses DBSCAN to detect values that are outside the normal range in the weaving-area-specific feedback current data as outliers and compares the number of these outliers for each left and right area. As the distance between the torch and the workpiece narrows, the feedback current values become unstable, and sometimes control of short-circuit transition occurs. These conditions should be detected as outliers through clustering. In other words, the area where outliers are detected more frequently is considered to be closer to the workpiece, and by applying an offset in the opposite direction of that area, highly responsive seam tracking can be performed.

It should be noted that applying the proposed technique to the condition described in Section 3.1 does not identify and avoid the unstable measurements when the gap is located at the weaving stay point since this phenomenon occurs due to the slow response speed of seam tracking. Therefore, by applying the proposed technique, the response speed for detecting curvature can be increased through control based on outlier detection, which enables the quick avoidance of the example condition itself.

Since the proposed technique is based on DBSCAN, the values of eps and minPts must be set according to the distribution and characteristics of the data to effectively detect the appropriate outliers. In a structured and stable welding environment, the range of current values is constant and the measurement cycle is stable, so the appropriate eps value can be determined through experimentation. However, in actual welding environments, the range and distribution of current values change dynamically due to the conditions of the workpiece and the changes in the distance between the torch and the workpiece, and time intervals between the collected current values are irregular due to unstable measurements. Therefore, in order to detect the appropriate outliers in actual welding environments, it is necessary to find an eps value that can quickly respond to dynamic conditions rather than using a fixed eps value.

In the proposed technique, the eps value is newly searched for each weaving cycle that includes the left and right weaving areas once, and this eps determination process is executed every half cycle of weaving; that is, after deriving the left and right areas of cycle 1, the process next derives them for the right area of cycle 1 and the left area of cycle 2, and then searches for the left and right areas of cycle 2. By dynamically determining eps using only the current input values in this way, the responsiveness of seam tracking can be enhanced.

Algorithm 1 shows the process of determining eps based on the median value for each of the two weaving areas and the procedure for applying MBSC based on that. The MBSC procedure takes the two target areas and the minPts value as input, calculates the range of current values $r_{L},r_{R}$ for each area, and then applies minmax scaling to normalize all values to be between 0 and 1. After that, appropriate eps values, $\varepsilon_{L}$ and $\varepsilon_{R}$, are calculated for each area. First, in lines 18 and 19, the median current value *m* is found and all points with current values within 5% of this median are added to the median points group $P_{m}$. In lines 20 to 26, for each median point, the distance to all points within the area is calculated, and then the average distances to the minPts−1th nearest points $d_{k}$ are calculated. For example, if minPts is 5, it calculates the average distance to the fourth most distance point for all points in the median points group. This average value is determined as eps for that area. Afterwards, from lines 7 to 11, scaling operations are performed on eps based on the range of current values for each area and, finally, clustering is performed using the determined eps and minPts.
**Algorithm 1** Determination of eps using median and MBSC   **Input:**   $P_{L},P_{R}\leftarrow$ points as (time, current) pairs of current values measured from left and right weaving areas.   k← Minimum number of neighbors required to form a cluster (minPts).   **Output:**   $P_{L}^{*},P_{R}^{*}$   // $P_{L}$ and $P_{R}$ with normalization and labeling performed.1:**procedure** MBSC($P_{L},P_{R},k$)2:   $r_{L}\leftarrow \max\left(C_{L}\right)-\min\left(C_{L}\right),$   $C_{L}=\left\{c|\left(t,c\right)\in P_{L}\right\}$3:   $r_{R}\leftarrow \max\left(C_{R}\right)-\min\left(C_{R}\right),$   $C_{R}=\left\{c|\left(t,c\right)\in P_{R}\right\}$4:   $P_{L}^{\prime },P_{R}^{\prime }\leftarrow$ Perform minmax scaling on $P_{L},P_{R}$ to make the values range from (0,0) to (1,1).5:   $\varepsilon_{L}\leftarrow \mathrm{CalculateEps}\left(P_{L}^{\prime },k\right)$6:   $\varepsilon_{R}\leftarrow \mathrm{CalculateEps}\left(P_{R}^{\prime },k\right)$7:   **if** $r_{R}>r_{L}$ **then**8:      $\varepsilon_{R}\leftarrow \varepsilon_{R}\cdot \left(r_{R}/r_{L}\right)$9:   **else**10:      $\varepsilon_{L}\leftarrow \varepsilon_{L}\cdot \left(r_{L}/r_{R}\right)$11:   **end if**12:   $P_{L}^{*}\leftarrow \mathrm{DBSCAN}\left(P_{L}^{\prime },\varepsilon_{L},k\right)$13:   $P_{R}^{*}\leftarrow \mathrm{DBSCAN}\left(P_{R}^{\prime },\varepsilon_{R},k\right)$14:   **return** $P_{L}^{*},P_{R}^{*}$15:**end procedure**16: 17:**function** CalculateEps($P^{\prime },k$)18:   $m\leftarrow \mathrm{median}(\left\{c|\left(t,c\right)\in P^{\prime }\right\})$19:   $P_{m}\leftarrow \left\{\left(t,c\right)\in P^{\prime }|m\times 0.95\leq c\leq m\times 1.05\right\}$20:   d←021:   **for all** $\left(t_{i},c_{i}\right)\in P_{m}$ **do**22:      $D\leftarrow \{\sqrt{{\left(t_{i}-t\right)}^{2}+{\left(c_{i}-c\right)}^{2}}|\left(t,c\right)\in P^{\prime }\}$23:      Sort ascending *D* and let $d_{k}\leftarrow D\left[\left(k-2\right)\right]$24:      $d\leftarrow d+d_{k}$25:   **end for**26:   $\varepsilon =\frac{d}{|P_{m}|}$         ▹ Calculate the average of the (k−1)-th distances27:   **return** ε28:**end function**

The reason for preferring the median as a standard is that, even when the current values within a certain weaving areas are constant or show large amplitudes up and down, the median is likely to represent the normal current values in the area. Additionally, to maximize clustering formation and outlier detection ability, the minimum eps value appropriate for the given minPts is determined. If eps is too large compared to the level of minPts, there is a high likelihood that outliers will be included in other clusters, and if too small, many outliers may occur. Generally, for 2D data, it is recommended to set the minPts value to 4 to balance the cluster formation and outlier detection performance. However, in this study, since the main purpose is to detect outliers in the feedback current, minPts is set to 6 [32]. The eps value is set as the distance to the minPts−1th closest point from the median point, which ensures that the median point always belongs to a cluster.

With the minPts value set as somewhat large, the process of determining the eps value becomes easier due to the influence of outliers, and the eps value can be greatly affected by the time index of the median. For example, if the point selected as the median is close in time index to an outlier, the likelihood increases that outliers will be included among the points up to minPts−1. Alternatively, if the median point is located in a region with a sparse time index, an eps value could be derived that is large enough to include outliers in a cluster. Therefore, to reduce the influence of the median point’s time index, the Euclidean distance to the minPts−1th nearest point is averaged using additional data within an appropriate range from the median, and this average is used as the eps value to decrease the possibility of distortion. At this time, when selecting the median point group, setting a range too narrow, around ±1% to 2% of the median, does not sufficiently correct the distortion due to the median index. On the other hand, setting too wide a range, around ±10%, reduces the outlier detection performance. Hence, a ±5% range is set as appropriate. This range can vary depending on the weaving speed or sampling cycle.

The proposed technique applies DBSCAN based on the appropriately determined eps value to detect outliers in each of the left and right weaving areas, and compares the number of outliers in each area to apply an offset to the torch based on this comparison. However, since data normalization is performed for each area, there arises a problem where the scale of the base data from which each eps is derived as different. Therefore, to scale eps, it is necessary to adjust the eps value using the range of feedback current value changes in each area before performing DBSCAN. For example, a smaller range of current change means that the data are clustered near the normalized median value, and, in this case, it is desirable to detect fewer outliers compared to other areas. Accordingly, the eps value for the area with a smaller range of current change is increased proportionally compared to the area with a larger range, and then outliers are detected.

Algorithm 2 shows the process of performing seam tracking based on the outliers detected in each weaving area by the MBSC algorithm. Since the frequency of outliers is high in areas where the instability of the feedback current is relatively high, the position of the torch can be corrected and the weld seam can be accurately tracked by applying an offset in the opposite direction of the area. This approach enhances efficiency by enabling quick seam tracking even in conditions where the existing arc-sensing technique, which is based on the average current of each area, cannot effectively detect the torch’s bias due to the control of short-circuit transitions. In addition, if the frequency of outliers is similar in both areas, the existing arc-sensing technique, which performs seam tracking using the average feedback current, is applied to adjust the position of the torch more frequently. The difference in outlier frequency and the offset in MBSC-based seam tracking should be appropriately set according to the welding environment, and this approach improves the precision of seam tracking.
**Algorithm 2** MBSC-Based ArcSensing   **Input:**   $P_{L}^{*},P_{R}^{*}\leftarrow$ labeled current values including clustering result from MBSC.   l← current location of the welding torch.   Δ← offset for welding torch movement per iteration.   t← threshold for outlier ratio to decide movement.   **Output:**   $l^{\prime }$   // updated location of the welding torch.1:**procedure** MBSC-BasedArcSensing($P_{L}^{*},P_{R}^{*},l,\Delta ,t$)2:   $o_{L}\leftarrow \left|\left\{p\in P_{L}^{*}:p\;\mathrm{is}\;\mathrm{labeled}\;\mathrm{as}\;\mathrm{outlier}\right\}\right|$3:   $o_{R}\leftarrow \left|\left\{p\in P_{R}^{*}:p\;\mathrm{is}\;\mathrm{labeled}\;\mathrm{as}\;\mathrm{outlier}\right\}\right|$4:   **if** $o_{R}\neq 0$ **and** $o_{L}/o_{R}>t$ **then**5:      $l{\;}^{\prime }\leftarrow l-\delta$                ▹ Move torch left6:   **else if** $o_{L}\neq 0$ **and** $o_{R}/o_{L}>t$ **then**7:      $l{\;}^{\prime }\leftarrow l+\delta$               ▹ Move torch right8:   **end if**9:   **return** $l{\;}^{\prime }$10:**end procedure**

### 3.3. Application of the MBSC Algorithm Based on Actual Data

**Figure 6:**
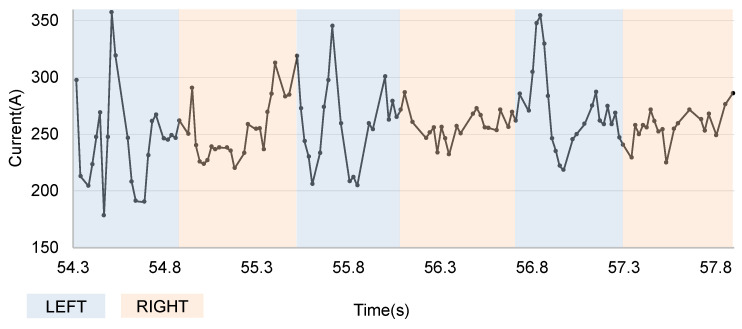
Classification of normal range current data by each weaving area.

In this section, we explain in detail how the proposed algorithm works using actually measured feedback current data. The data used are as shown in Figure 6, which shows an instance where a left bias occurs in conditions where a gap exists between curved workpieces and the weaving stay point is just before reaching the gap. If this bias is not detected and the weaving stay point reaches the gap, the feedback current pattern becomes like that shown in Figure 3b, making it difficult to normalize the welding operation with arc sensing alone. In conclusion, it is difficult for arc sensing based on weaving area average values to quickly respond to such bias in challenging and unstructured environments, leading to the failure of automatic welding. On the other hand, the proposed technique can effectively detect outliers even in these highly changing patterns, increasing the probability of successfully performing seam tracking.

**Figure 7:**
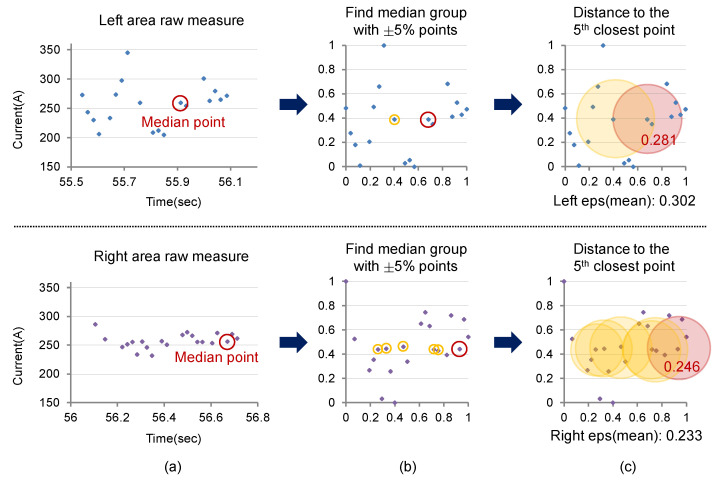
Weaving- area-specific normalization and eps determination process: (**a**) Weaving-area-specific measured feedback current values; (**b**) Results of normalization and finding the median group; (**c**) Determination of eps using the 5th closest point of the median group.

Figure 7 shows the process of determining eps for the left and right weaving area current data of the second weaving cycle seen in Figure 6. After normalizing the current data for each area, the median point based on the current values is found. At this time, the current value of the median point of each area can be considered to represent the current value of that area. (For intuitive confirmation, the leftmost graph shows the raw input values before normalization, but, in reality, the median point is found after normalization.) It can be observed that the median point of the right area is biased to the right. If we try to calculate eps only at this point, the result may be distorted by the time index. Therefore, data within ±5% of the current value of the median point are selected as the median points group. Afterwards, the Euclidean distance to the minPts−1th closest point for each point in this group is calculated, and this is averaged to determine the eps value. For the left area in the figure, the distance to the minPts−1th closest point from the median point is 0.281, but the arithmetic mean of the median points group is 0.302, so this value is determined as eps for that area. The arithmetic mean of the distances to the minPts−1th closest point for each point in the median points group of the right area is 0.233, so this value is determined as eps.

Next, it is necessary to match the reference scales of eps calculated in the two weaving areas. The range of current values in the left area, $r_{L}$, is 345.0−204.6=140.4, and $r_{R}$ in the right area is 286.5−231.9=54.6. Since the range of current values in the right area is relatively small, it can be said that the current values in the right area tend to be more stable. Therefore, considering the scale of the left area, eps should be scaled up in the right area to detect outliers. Thus, the eps for the right area is finally determined as (140.4/54.6)×0.233=0.599.

**Figure 8:**
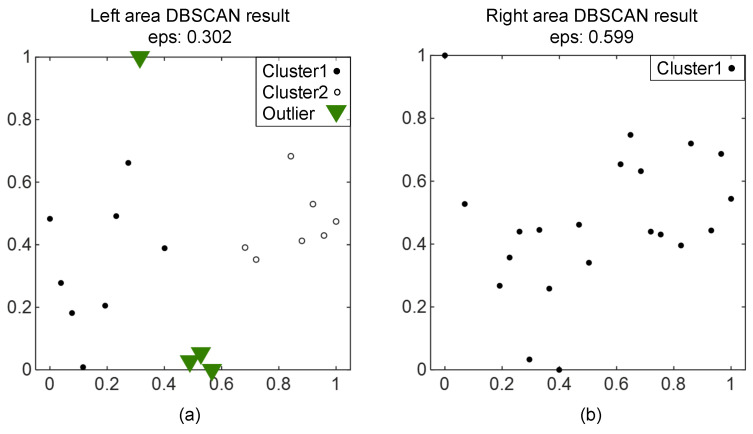
Feedback current data with the MBSC algorithm applied: (**a**) the result of DBSCAN performed in the left weaving area with eps=0.302,minPts=6; (**b**) the result of DBSCAN performed in the right weaving area with eps=0.599,minPts=6.

Figure 8 shows the results of performing DBSCAN for each weaving area using the eps derived from the previously performed eps determination process. The number of groups is ignored, and four outliers were detected in the left area and no outliers were detected in the right area. This means that more unstable feedback current values were measured in the left area compared to the right area, suggesting that the torch is closer to the workpiece in the left area when weaving. Therefore, by applying the proposed technique, an offset can be applied to move away from the left area based on these results. On the other hand, using average-value-based arc sensing on the same dataset would not be able to respond quickly to such unstable current value patterns.

**Figure 9:**
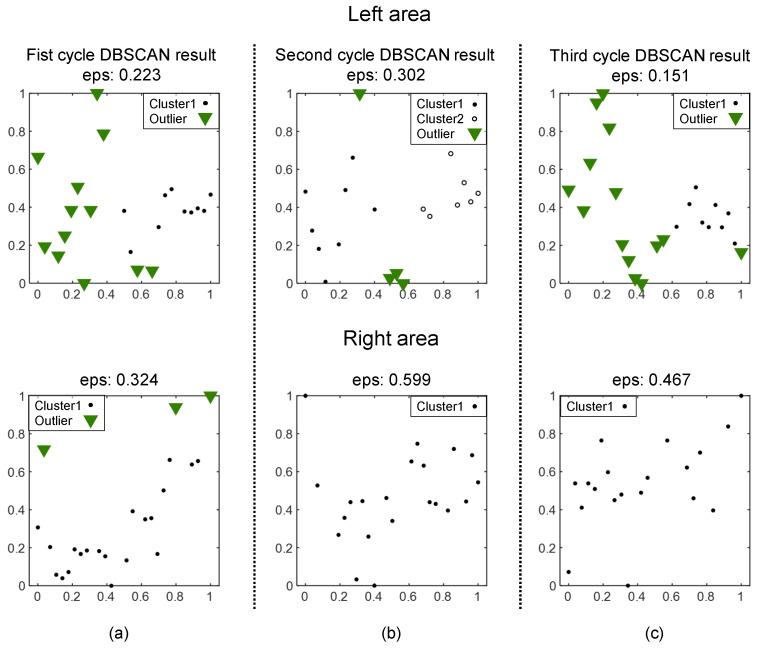
Results of applying the MBSC algorithm to the data in Figure 6: (**a**) MBSC results for the first weaving cycle; (**b**) results for the second cycle; (**c**) results for the third cycle.

The MBSC results for different weaving areas of Figure 6 are shown in Figure 9 and Table 1. In Figure 9, it can be seen that parts of the sudden increases and decreases in current values (left area of the second cycle) as well as the entirety (left areas of the first and third cycles) are detected as outliers. In Table 1, it can be seen that outliers are appropriately detected even when determining eps every half cycle. The proposed technique has thus been demonstrated to effectively respond to conditions with large-curvature workpieces or gaps in certain levels of curvature, such as unstructured environments where unstable current values are observed. In the next section, we will analyze in detail the applicability and performance of the proposed technique through experiments targeting various real unstructured environments.

## 4. Experiment Result

### 4.1. Experiments on Workpieces with Small Curvature

**Figure 10:**
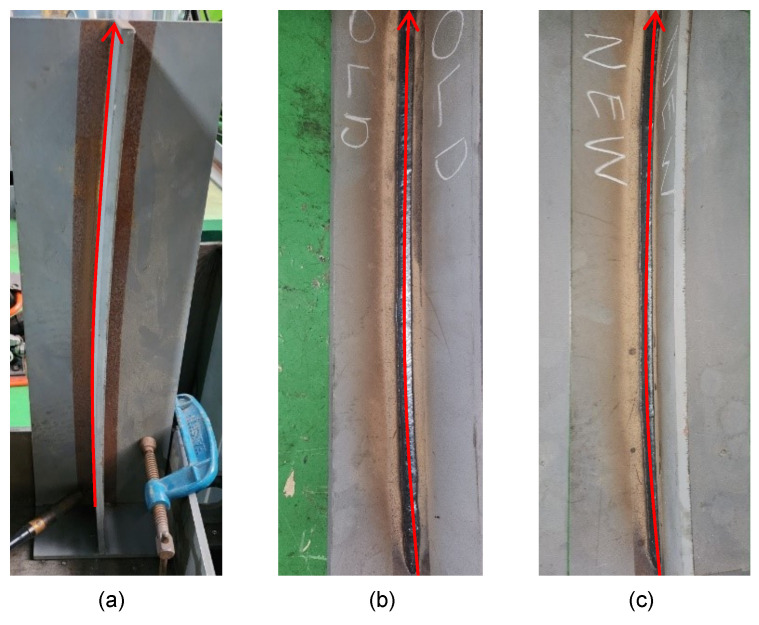
Welding results for the R4000 workpiece: (**a**) curved workpiece with a radius of 4000 mm, (**b**) arc-sensing welding results based on average current, (**c**) arc-sensing welding results based on MBSC.

In this study, we evaluate the seam tracking performance by comparing the existing average-value-based arc-sensing technique with the MBSC-based arc-sensing technique under various welding environments. For all experiments, the use of external measuring devices such as oscilloscopes causes severe changes to existing control systems, so a current value measurement was performed using Hall sensors attached to torches with comparable accuracy. While using straight workpieces and changing their direction during welding is a common method for evaluating the accuracy of arc sensing, this method makes it difficult to precisely measure the degree of distortion in the comparison between the two techniques. Therefore, in this experiment, we conduct a performance comparison between the two techniques on workpieces that include curvature, starting with workpieces that have a small curvature. These workpieces have a curvature of a radius of 4000 mm (R4000), and the welding quality is evaluated by comparing the torch movement trajectory between the two techniques under the same conditions. The experiment was conducted on a welding length of 600 mm, and the results are shown in Figure 10 and Table 2.

**Figure 11:**
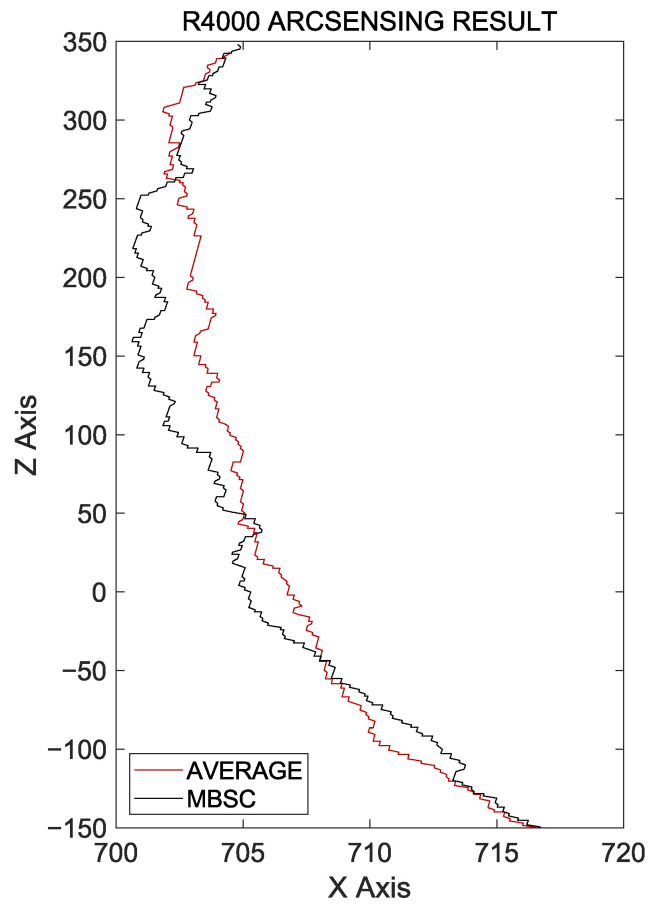
Torch coordinates during welding operations on the R4000 workpiece.

In this experiment, both automatic welding using the existing arc-sensing technique and the MBSC-based arc-sensing technique was successfully completed within the normal range. However, when calculating the radius of the torch movement trajectory using the ordinary least squares (OLS) method based on the data presented in Figure 11, the arc-sensing technique based on the average current failed to quickly follow the curvature of the workpiece, resulting in a measurement of 5400.1 mm. On the other hand, when the MBSC technique was used, the result was 3616.4 mm, which is much closer to the actual curvature of the workpiece. This indicates that the MBSC-based arc-sensing technique tracks the weld seam more quickly and precisely than existing technique.

### 4.2. Experiments on Workpieces with Large Curvature

In this study, additional experiments were conducted using a workpiece with a relatively large curvature with a radius of 2000 mm. Additionally, to provide a more challenging environment, the direction of the robot’s movement was intentionally adjusted, and the position of the welding endpoint was changed to ensure that the welding path detected by arc sensing was continuously biased. Like the first experiment, this experiment also involved a total welding length of 600 mm.

**Figure 12:**
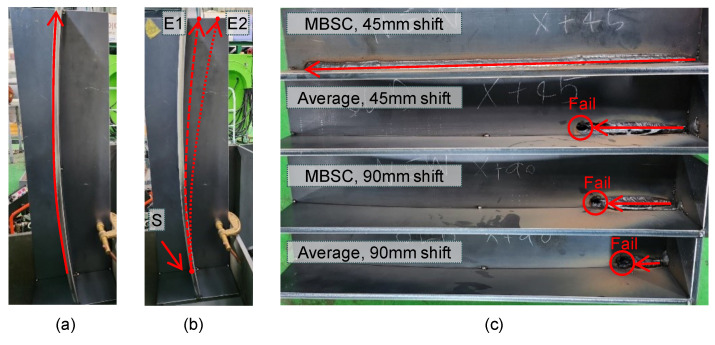
(**a**) Curved workpiece with a radius of 2000 mm, (**b**) new endpoints E1 and E2 shifted by +45 mm and +90 mm, respectively, from the welding start point S and the original endpoint, (**c**) photos of completed welding for each case.

**Figure 13:**
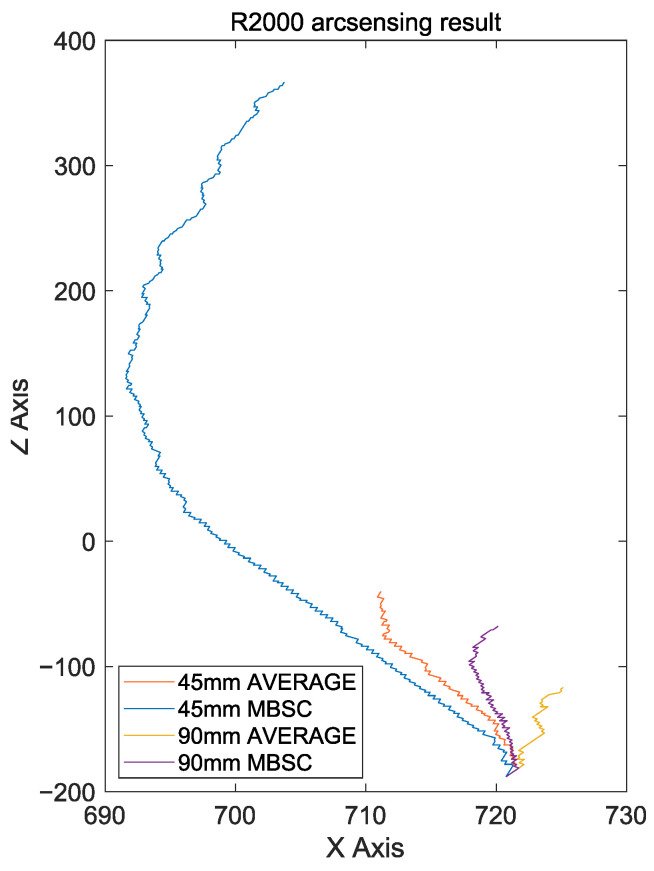
Torch coordinates during welding operations on the R2000 workpiece.

Figure 12b shows the start and endpoints of the welding path. E1 represents the position where the endpoint of the welding path is moved +45 mm from its original position. This change causes the robot to try to move from S to E1, resulting in a continuous bias. In other words, even if the robot performs normal seam tracking, this setup can show a persistent bias, partially simulating a scenario where the welding robot’s articulation becomes loose. E2 represents the position where the endpoint of the welding path is moved +90 mm from its original position, similarly prompting the welding robot to aim toward moving from S to E2. Figure 12c and Figure 13 show the welding results for each case.

When the endpoint was moved by 45 mm, the existing arc-sensing technique performed seam tracking well in the beginning but missed track of the weld seam at a certain point and became biased. The spot marked as “Fail” shows where the torch became biased and caused a puncture in the workpiece. On the other hand, the MBSC-based arc-sensing technique was very effective at performing seam tracking, even though the path was set to be biased in the workpiece with high curvature. The proposed technique continuously detected outliers and quickly responded to bias in the weld seam, ensuring that the welding baseline was not missed until the end.

When the endpoint was moved by 90 mm to intentionally bend the path by about 8.5 degrees, both arc-sensing techniques observed a seam tracking failure. The existing arc-sensing technique completely failed to control the torch’s bias, and welding was stopped at the 71 mm point. On the other hand, the MBSC-based arc-sensing technique initially showed results that tracked the weld seam to some extent, but it stopped welding at the 120 mm point. This is believed to be due to a limitation of the maximum offset that can be adjusted per unit time, leading to the weld seam being lost.

### 4.3. Experiments on Curved Workpieces with Gaps

**Figure 14:**
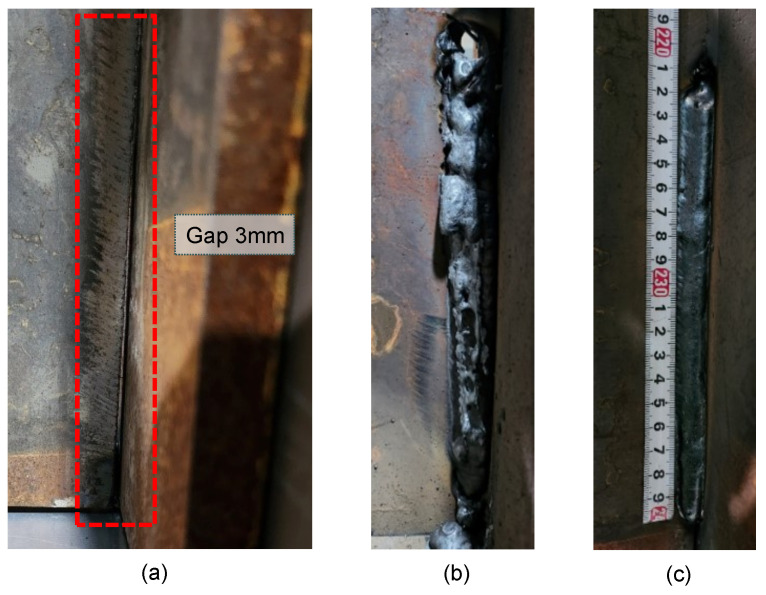
(**a**) Workpiece with a 3 mm gap, (**b**) results of existing arc sensing, (**c**) results of MBSC-based arc sensing.

In the third experiment, the test was conducted assuming the existence of gaps in the curved workpiece. As shown in Figure 14a, a gap of 3 mm was intentionally left between the workpieces. Additionally, similar to the second experiment, the welding endpoint was shifted to create a challenging condition where the weld seam was continuously biased. The experiment was conducted over a total distance of 200 mm.

**Figure 15:**
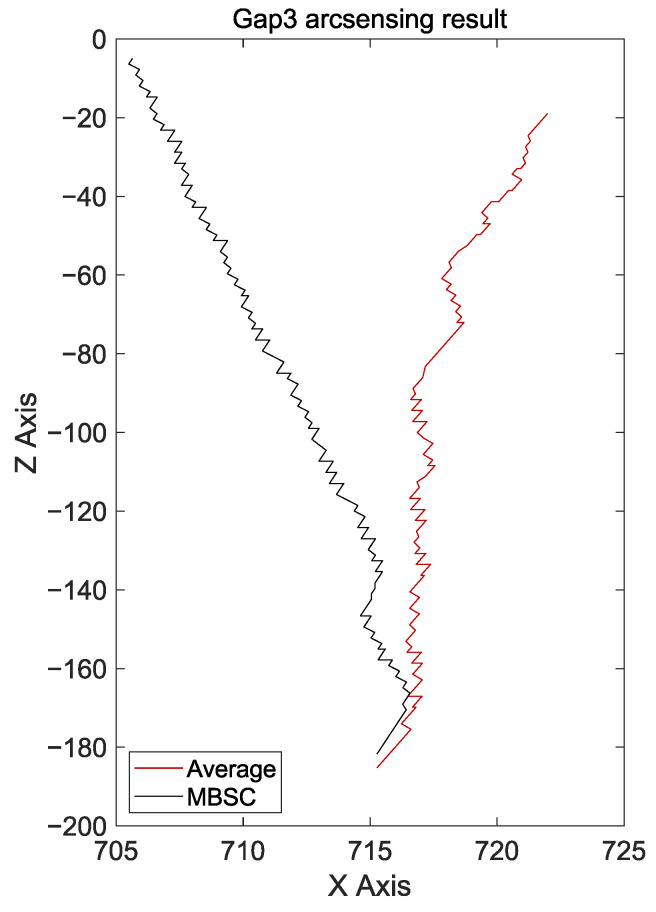
Torch coordinates during welding operations on workpieces with gaps.

When applying the existing arc-sensing technique, seam tracking showed high instability due to the inability to quickly detect bias in the weld seam. As a result, as seen in Figure 14b, the weld bead was roughly formed. Moreover, as shown in Figure 15, seam tracking ultimately failed and became biased. This can be attributed to the fact that, when sudden increases and decreases in feedback current at the weaving stay point, where a gap is located, are repeated, recovery through arc sensing alone becomes impossible. On the other hand, when the MBSC-based arc-sensing technique was used, normal welding was performed as shown in Figure 14c. This result proves that the proposed method can perform highly responsive seam tracking, ensuring that gaps do not match up with the weaving stay points, even in conditions of continuous bias.

## 5. Discussion

The results for the three experiments in this study clearly show that the MBSC algorithm performs well for workpieces with small curvature, workpieces with large curvature, and workpieces with gaps. These results prove that the proposed technique, which adjusts the torch bias by detecting the outliers of the feedback current, adjusts the bias of torch more quickly and effectively than the existing arc-sensing technique, which uses the average of the feedback current. Applying MBSC-based seam tracking to welding robots is expected to enable welding automation for workpieces in various welding environments.

However, the result of the second experiment showed that seam tracking failed when the welding path was wrong and the curvature of the workpiece was very severe. This is thought to be due to the possibility of distortions arising from corrections in robot systems with low reduction ratios or due to aging equipment that makes fine control difficult. These limitations can be addressed by applying data analysis techniques that can more accurately reflect challenging welding environments and improving the maximum value of offset adjustments for each weaving cycle.

MBSC-based seam tracking requires only a relatively inexpensive Hall sensor, which is more cost-effective than vision sensors or 2D laser sensors considered for seam tracking. In addition, while vision sensors consume about 10 W of power and 2D laser sensors require an additional 12 W of power on average, Hall sensors consume no power because they convert incoming current data into analog signals. This makes the MBSC-based seam tracking technique more economical than other techniques that utilize additional sensors.

## 6. Conclusions

This study focused on the problem of the reduced reliability of the feedback current due to control of short-circuit transition, occurring in conditions where the distance between the torch and workpiece rapidly decreases due to the severe curvature of the workpiece or where gaps exist between curved workpieces. An unstable feedback current leads to a failure in the weaving-area-average-based arc-sensing technique to quickly correct torch bias, resulting in seam tracking failure and, consequently, failed welding operations. Accordingly, this study proposed an MBSC technique based on DBSCAN. The proposed technique performed seam tracking by detecting outliers through determining an eps value centered around data near the median of the feedback current at every half cycle of weaving. This approach quickly corrects the torch position when seam bias occurs, enabling highly responsive seam tracking.

The proposed technique was verified through actual welding experiments. Compared to the existing arc-sensing technique, the proposed technique tracked the weld seam more precisely in workpieces with small curvature and successfully performed welding even in conditions of continuous seam bias in workpieces with large curvature. Additionally, in cases where gaps existed between workpieces, MBSC-based arc sensing completed the welding successfully, while the existing technique failed to perform seam tracking correctly due to the influence of the gaps.

Also, the proposed technique performs quick and accurate arc sensing even in conditions where feedback current values are unstable or when control of short-circuit transition occurs. It has proven its effectiveness in challenging automatic welding tasks that reflect actual environments with large curvature or gaps. However, the technique has limitations in that it assumes generalized geometric parameters of the weld, such as bead size and surface characteristics. In the future, we intend to utilize the established welding robot testbed to study clustering techniques for arc-sensing automated welding that can consider a variety of geometric parameters and environmental conditions, and to continuously improve and adapt this technique for a wider range of challenging actual welding environments. Ultimately, our goal is not only to perform seam tracking but also to contribute to the improvement of welding quality.

## Figures and Tables

**Table 1:** Results of applying the MBSC algorithm to the data in Figure 6.

Comparison Target Weaving Areas	Weaving Area	Scaled Eps	Outliers
1L1R	1L	0.223	12
	1R	0.324	3
1R2L	2L	0.302	4
	1R	0.255	3
2L2R	2L	0.302	4
	2R	0.599	0
2R3L	3L	0.151	14
	2R	0.580	0
3L3R	3L	0.151	14
	3R	0.467	0

**Table 2:** Radius of the workpiece calculated using the torch coordinates.

Technique	Result of OLS
Arc sensing based on average current	5400.1 mm
Arc sensing based on MBSC	3616.4 mm

## Data Availability

The original contributions presented in the study are included in the article, and further inquiries can be directed to the corresponding author.
